# Factors Influencing Physical Activity Engagement Amongst Those Living With Pulmonary Hypertension in the UK

**DOI:** 10.1002/pul2.70332

**Published:** 2026-06-11

**Authors:** Sarah J. Hardcastle, Zachary Wells, Carol Keen, Robin Condliffe, David G. Kiely, Ciara McCormack

**Affiliations:** ^1^ School of Sport and Physical Activity Sheffield Hallam University Sheffield UK; ^2^ Sheffield Teaching Hospitals NHS Foundation Trust Sheffield UK; ^3^ Sheffield Pulmonary Vascular Disease Unit, Royal Hallamshire Hospital Sheffield Teaching Hospitals NHS Foundation Trust Sheffield UK; ^4^ NIHR Biomedical Centre Sheffield Sheffield Teaching Hospitals Sheffield UK; ^5^ Department of Sport Science and Nutrition Maynooth University Kildare Ireland

**Keywords:** behaviour change, exercise, lifestyle, motivation, qualitative

## Abstract

This study explored attitudes towards exercise, recollections of physical activity advice received, and the dimensions that influence physical activity (PA) engagement in individuals diagnosed with pulmonary hypertension. Virtual semi‐structured interviews (*n* = 21) were conducted with those diagnosed with pulmonary hypertension. Participants (mean age 57.4 (SD ± 13.1) were recruited through the Pulmonary Hypertension Association of the UK. Interviews were transcribed verbatim and analysed using reflexive thematic analysis. Four main themes were generated: (i) Fear of breathlessness and overdoing it; (ii) lack of motivation and desire for monitoring and targets; (iii) self‐presentation: keeping up appearances, and (iv) Little PA advice: patient‐driven communication. A lack of motivation was a common barrier to PA engagement amongst PH patients. Other dimensions included fear and anxiety related to dyspnoea and overexertion and protective self‐presentation. The influence of self‐presentation and identity in exercise‐avoidance in PH is a novel finding. The study also found recollections of little PA advice from treating clinicians and a tendency for patient driven PA information. Future PA interventions that alleviate fear and anxiety through cognitive restructuring, provide reassurance on the safety and benefits of exercise, and include clear instruction on behaviour and graded exposure would be worthwhile to increase PA in PH. Interventions that broaden the conception of PA and promote lifestyle‐based PA alongside fostering patient motivation for PA through goal setting, self‐monitoring and review of behaviour are also likely to be valuable.

Pulmonary Hypertension (PH) is a rare and incurable cardio‐respiratory disease that regardless of aetiology, gives rise to a range of debilitating symptoms including dyspnoea, fatigue, palpitations and dizziness that compromise physical function and health related quality of life (HRQoL) [1]. As such, daily physical activity (PA) is often compromised among those living with PH, with lower activity levels associated with reduced exercise capacity and poorer survival [2, 3].

According to international recommendations, all adults should undertake at least ≥ 150 min/week of moderate‐intensity PA or ≥ 75 min/week of vigorous‐intensity PA, or an equivalent combination of the two (≥ 150 min/week of moderate‐to‐vigorous PA (MVPA) for improved health [4, 5]. However, few PH patients achieve the PA guidelines and most tend to be highly sedentary [2, 3, 6]. There is increasing evidence to suggest that insufficient PA and prolonged inactivity in patients with PH may further compromise aerobic capacity, hemodynamic function and the odds of survival [2, 7]. Increased PA is associated with improved physical function and HRQoL in those with pulmonary arterial hypertension and chronic thromboembolic pulmonary hypertension [2, 8]. Further, meta‐analyses confirm that exercise engagement significantly improves exercise capacity, cardiorespiratory fitness and HRQoL among patients with PH and exercise has been proven to be safe and well tolerated for stable patients with PH [9, 10, 11].

Therefore, given the role of PA to improve exercise capacity, HRQoL and survival and the low levels of PA amongst those living with PH, there is a need to better understand attitudes toward PA and the dimensions influencing exercise engagement to design appropriate and patient‐centred PA interventions. There is a relative dearth of research concerning the factors influencing exercise engagement in PH and most have focused on the barriers to PA amongst those living with PH using a survey design [12, 13, 14]. In these studies, motivational barriers such as a ‘lack of will' and lack of self‐discipline or a lack of interest have been identified as PA barriers for this population [12, 14]. Fear and uncertainty regarding the safety and effectiveness of exercise have also been identified as barriers to exercise engagement for those living with PH alongside dyspnoea, fatigue, and anxiety [13]. The only qualitative study in this area has extended our understanding of the nature of fear of exercise and how it influences exercise engagement amongst PH patients (i.e., in that fear of exercise can be due to perceived overexertion, damage to health or breathlessness or combination of these) [15]. A further qualitative study exploring PA knowledge and support needs in Ireland found that PH clinicians provide suboptimal PA advice, yet participants expressed a strong desire to receive such guidance and support from their treating clinicians [16].

Qualitative approaches are useful to capture the range of influences on behaviour and offer an in‐depth perspective on individuals' perceptions and experiences that may help to identify the salient dimensions that influence PA engagement. To our knowledge, no prior research has explored PA advice received from treating clinicians or the dimensions influencing PA engagement amongst those living with PH residing in the UK. Therefore, the aim of the present study was to explore attitudes towards exercise, recollections of PA advice received from treating clinicians, and the dimensions that influence PA engagement in individuals diagnosed with PH.

## 1 Methods

### 1.1 Participants and Procedure

Eligible participants were adults diagnosed with pulmonary hypertension and resident in the UK. Participants were recruited through an advert on social media channels of the Pulmonary Hypertension Association (PHA) UK (Facebook, Instagram and X) or through a formal invitation email from the research team disseminated via the PHA UK to a list of subscribed members (Research Forum) where it is known whether the member is a PH patient (*n* = 39). The email included a brief description of the study and a participant information sheet. Following an expression of interest, name and preferred contact details were forwarded from the PHA to the research team to make direct contact with the participant. A research assistant contacted participants who expressed a willingness to participate, and an interview date was arranged. Participants provided written, informed consent prior to the interview and were informed that pseudonyms would be used in any reporting of the data. Participants also completed a sociodemographic questionnaire to report age, marital status, highest educational attainment, gross household income, postcode (to identify location and index of multiple deprivation), PH World Health Organisation (WHO) group, comorbidities and HRQoL. Comorbidity was assessed using the Self‐administered Comorbidity Questionnaire [17]. The Self‐administered Comorbidity Questionnaire includes twelve medical conditions, and participants have the option of indicating three additional conditions. Up to 3 points are allocated to each condition (i.e., one for the condition, one for the treatment, and an additional point if the condition causes a limitation in functioning, with a maximum score of 36). HRQoL was assessed using the emPHasis‐10 [18], consisting of ten questions using a 6‐point scale (0‐5) for a total maximum score of 50 (the higher the score, the worse the quality of life). At the point of the interview, participants were asked to self‐report their WHO functional class, if known. The current study conformed to the Standards for Reporting Qualitative Research (online Supplemental File 1) [19]. Sheffield Hallam University Research Ethics Committee approved this study (Ref: ER50098142).

Semi‐structured online interviews (mean duration = 49.2 min ± 7.6) between April 2023 and October 2024 were conducted by SH or CM or both. The lead author (SH) has a wealth of experience in qualitative data collection and analysis, and in PA behaviour change and health psychology. CM also has qualitative research experience with specialist knowledge in PH and exercise physiology. An interview guide (online Supplemental File 2) was developed based on previously published work [15], with questions concerning PH diagnosis, PA history, PA barriers, influences on PA, exercise preferences and recollections of PA advice or support from PH clinicians. The interviews were digitally recorded and transcribed verbatim.

### 1.2 Data Analysis

The data was manually analysed by the first author using reflexive thematic analysis to generate themes [20]. Analysis included deductive and inductive approaches; whist a codebook was not adopted, and categories were not pre‐determined, it is recognised that the interview guide focused on the dimensions influencing PA including clinician‐delivered PA advice and therefore analysis was not entirely inductive. Nevertheless, data was ‘open‐coded' to best represent the perceptions and experiences (in relation to the research aims) as conveyed by participants [21]. Thematic reflexive analysis involved several steps including (i) *immersion* and the careful reading of transcripts; (ii) attaching codes to salient text segments; (iii) the identification of themes at a broader level and examining whether codes may be combined to form an overarching theme. During these processes, inductive analysis was used to generate themes grounded in the data. Although it is recognised that data interpretation may be influenced by the researcher's prior knowledge, an attempt was made to be open to new findings that may conflict with theory and previous research findings [22, 23]. The final step involved reviewing themes, cross‐checking for overlap, and finally defining and classifying themes. The analysis offered is one interpretation of the data and other interpretations are possible. Nevertheless, we aim to offer a trustworthy interpretation that accurately captures the data and use ‘thick description' with extensive quotations so that the reader can judge the interpretations [24].

## 2 Results

Forty‐eight individuals expressed an interest to participate in the study and provided contact details that were forwarded to the research team. Sixteen did not reply to initial emails from the research team. Two withdrew because their initial PH diagnosis was incorrect and another withdrew due to concerns regarding online security. One participant arranged an interview but could not attend. A further seven expressed an interest to participate beyond the data collection period. Twenty‐one participants completed an interview (mean age 57.4 ± 13.1 years). Table 1 displays participant demographics. Most (*n* = 15;71%) were female and diagnosed with pulmonary arterial hypertension (*n* = 11). Participants were recruited from across eight regions in England and Scotland and reported a compromised HRQoL evidenced by the mean Emphasis 10 score of 27.2 ± 13.5. Table 2 provides an overview of the individual characteristics of participants including age, location, PH type, WHO functional class and emPHasis 10 scores. Data analysis generated four main themes: (i) Fear of breathlessness and overdoing it; (ii) lack of motivation and desire for monitoring and PA targets; (iii) self‐presentation: keeping up appearances, and (iv) Little PA advice: patient‐driven PA guidance.

**Table 1:** Demographic characteristics of interview participants.

Age (years): mean ± SD	57.4 ± 13.1
*Sex: n (%)*	
Male: *n* (%)	6 (28.6)
Female: *n* (%)	15 (71.4)
*Ethnicity: n (%)*	
Caucasian	18 (85.7)
Indian	1 (4.7)
Other Asian	1 (4.7)
Other	1 (4.7)
*PH WHO group: n (%)*	
Group 1: PAH	11 (52.4)
Group 4: CTEPH	5 (23.8)
Group 5: Unknown cause	5 (23.8)
Duration of diagnosis (years): mean ± SD	6.8 ± 6.1
World Health Organisation Functional Class	2.5 ± 0.7
*Emphasis 10 QoL score (_/50): mean ± SD*	27.2 ± 13.5
< 16 (low risk)*	5 (23.8)
17‐33 (intermediate risk)	9 (42.9)
34 + (high risk)	7 (33.3)
SCQ score (_/45): mean ± SD	7.8 ± 7.5
Index of Multiple Deprivation: mean ± SD	7.0 ± 2.4
*Highest level of education: n (%)*	
Secondary school	2 (9.5)
Post‐school training/college/equivalent	6 (28.6)
University degree	12 (57.1)
Other qualifications	1 (4.8)
*Gross Household income: n (%)*	
< £10,000	1 (4.8)
£10,001 – £20,000	6 (28.6)
£20,001 – £35,000	4 (19.0)
£35,001 – £50,000	3 (14.3)
£50,001 – £65,000	3 (14.3)
£65,001 – £80,000	2 (9.5)
£80,001 +	2 (9.5)
*Marital Status: n (%)*	
Married	12 (57.2)
Divorced/separated	4 (19.0)
In a relationship	1 (4.8)
Single	4 (19.0)
*Geographical Region: n (%)*	
East Midlands	6 (28.6)
South East England	4 (19.0)
Greater London	2 (9.5)
North West England	2 (9.5)
Yorkshire & the Humber	2 (9.5)
Scotland	2 (9.5)
West Midlands	1 (4.8)
North East England	1 (4.8)
East of England	1 (4.8)

*Note:* * The Emphasis 10 thresholds of ⩽16, 17–33 and ⩾34) have been identified as distinct risk groups with significant survival differences (corresponding 1‐year mortality of 5%, 10% and 23%, respectively) (Lewis et al., 2021).

Abbreviations: CTEPH, chronic thromboembolic pulmonary hypertension; PAH, pulmonary arterial hypertension; PH, pulmonary hypertension; QoL, quality of life; SCQ, Self‐administered comorbidity questionnaire; SD, Standard deviation; WHO, World Health Organisation.

**Table 2:** Individual characteristics of participants.

ID code	Sex	Age	County	PH WHO group	Years since diagnosis	WHO FC	Emphasis 10 QoL score	SCQ score
01	F	69	Staffordshire	CTEPH	1	—	32	14
02	F	38	Greater London	Unknown	26	2	0	2
03	F	56	Scotland	PAH	4	4	38	10
04	F	42	Derbyshire	PAH	10	2	27	1
05	F	65	Derbyshire	Unknown	3	2	10	9
06	F	70	Merseyside	PAH	1	2	42	2
07	F	57	Northamptonshire	PAH	9	2	35	11
08	F	49	West Sussex	Unknown	11	2	22	1
09	F	66	Northamptonshire	CTEPH	6	—	26	13
10	F	62	Buckinghamshire	CTEPH	3	3	47	34
11	F	71	Cheshire	PAH	0	—	34	11
12	M	60	Scotland	PAH	6	3	32	3
13	F	67	South Yorkshire	Unknown	6	3	42	5
14	F	33	Essex	PAH	15	3	33	8
15	M	61	Lincolnshire	PAH	2	2	23	11
16	F	40	Tyne & Wear	PAH	9	2	25	7
17	M	62	West Sussex	PAH	5	2	14	7
18	F	57	West Yorkshire	PAH	8	4	48	—
19	M	76	Buckinghamshire	CTEPH	13	2	26	3
20	M	72	Middlesex	Unknown	1	2	14	2
21	M	34	Northamptonshire	CTEPH	3	2	2	2

Abbreviations: CTEPH, chronic thromboembolic pulmonary hypertension; F, female; M, male; PAH, pulmonary arterial hypertension; PH, pulmonary hypertension; QoL, quality of life; SCQ, Self‐administered comorbidity questionnaire; WHO FC, World Health Organisation Functional Class.

### 2.1 Fear of Breathlessness and Overdoing It

This theme was underpinned by a fear of exercise often stemming from breathlessness, fear of damage or simply fear of overdoing it and subsequent fatigue. Also, participant recollections of how PH was described to them caused fear. For example: “They described it as a rare and fatal disease…the exercise just dropped off and I was very frightened and scared” (P5, F, 65) and, “The fear comes from the fact they tell you it's a serious condition and listen to your body…if I exert myself and I can hear my heart then panic sets in” (P7, F, 57).

### 2.2 Fear Associated With Breathlessness

Most participants were fearful of exercise due to the increased breathlessness on exertion and this deterred exercise engagement: “It's knowing what pain is scary and what pain is good…I'd get out of breath going up the hill and is that good or bad pain? That put me off doing it…you don't know if it's heart pain or whether just you're unfit type of thing” (P10, F, 62), and, “I thought I was doing more harm than good when I was trying to walk and getting out of breath, and I could feel my heart racing. I hated it so I tended not to do anything” (P15, M, 61). Another participant echoed this point: “I got to the point where I didn't even want to go for a walk…It's the breathlessness when I get walking” (P5, F, 65). Another participant refers to fear of heart damage and how she has managed this PA barrier with encouragement from a friend: “If I'm pushing myself I'm doing it with fear because I'm thinking am I going to have a heart attack…I have a friend who is very encouraging and says 'It's doing you good, just stop, have a rest but keep going…when I get through those barriers I know I can do more so the next time it's like ignore the racing heart and breathlessness, just have a rest and keep on doing it” (P7, F, 57).

### 2.3 Fear of Overdoing It

For others, fear or anxiety concerning PA engagement was related to concerns of overdoing it (e.g., feeling faint or exhausted or subsequent fatigue that would put them out of action for a few days). For example, “There's fear as well…I don't know if I can do that big walk…what if I get to the top and feel really fatigued? I suffer a bit from anxiety …when I'm out for a walk and my heart is beating really fast, the anxiety can manifest a bit in terms of you can't do this. What if something happens to you? You're in the forest all by yourself” (P2, F, 38). The anxiety for this participant is partly linked to two episodes of fainting 6 years ago when running for a train and running up an escalator on the London underground “It goes back to when my confidence got knocked when I collapsed…now I feel like it sometimes holds me back a bit”. Others referred to fatigue: *“*I can't tell when I'm overdoing it so I'm not going to feel crushing exhaustion later on” (P18, F, 57), with fear of overdoing it preventing wanting to exert maximal effort when undertaking the 6MWT during routine clinic visit: “I purposely didn't go for 100% because what happens is I put everything into the 6 min and then I'm ill for 2 days” (P7, F, 57). Anxiety over capability and fears related to fainting or fatigue were echoed by other participants: “I was getting concerned about sitting all the time and thinking about doing things, and then I'd get up and it's all a bit I don't know if I can do it. Then I'd try to do it and I'm just wiped out” (P11, F, 71) and “social anxiety, the fear of passing out, and doing something new which is like going I really want to try and do that, but I don't know if it'll be good” (P16, F, 40).

Interestingly, despite the anxiety caused by breathlessness or concern of fainting, some participants recognised that the primary barrier is motivational: “It's partly a fear that has no real basis…I get frightened about going out…I've never been a person who has enjoyed going for a walk just for the enjoyment so I have to make a very conscious effort to do so…I suppose something goes off in my brain that says something might happen while you're out which it won't…I've proven it doesn't but that's in the back of my mind, it's an excuse really” (P1, F, 69) and “A lot of it is motivation. I know some of it is I feel a bit breathless today, I don't want to do that” (P5, F, 65). The former quote reveals how dimensions interact to influence PA engagement including issues related to fear, anxiety and confidence, in addition to a lack of enjoyment of exercise.

### 2.4 Lack of Motivation and Desire for Monitoring and PA Targets

Many participants identified a lack of motivation as the primary barrier to exercise engagement. For example, “I'm the biggest barrier because I've naturally been lazy all my life” (P15, M, 61) and “I'm very lazy…with me a lot of it is motivation” (P5, F, 65). There was also a recognition that sedentary behaviours are attractive due to being symptom free: “being sedentary for me is the most comfortable you can get with PH and that's the dilemma”(P17, M, 62). Some had participated in pulmonary rehabilitation programmes but tended to lack the motivation to continue and expressed a preference for group exercise due to poor motivation and self‐regulation: “I think group‐based thing for me personally if I'm at home on my own I know what I'm like…I'm a lazy shite” (P3, F, 56) and similarly “I wasn't really motivated to get on the bike myself (following participation in a research trial). Nobody's watching, nobody knows. But when you're part of a group exercise, then the motivation is there” (P7, F, 57). Others expressed a preference for home‐based exercise: “If they offered doing exercises in the gym I wouldn't do it because I want it to be adapted to my lifestyle…something like talk to somebody once a week and give me a routine…and I did that at whatever time of day I wanted to do it and then somebody chats to you once a week” (P4, F, 42) and “I don't think I could ever join a gym. I don't think I could do that, but I'll certainly be walking more and doing more exercise at home…like weights on my arms to get the muscles back…I need motivation to exercise, I always have” (P15, M, 61). A preference for remote or digital support was also identified. For example, “Something I could do remotely because I don't live close to the hospital where I have appointments” (P2, F, 38) and “An app so you can do it at home” (P16, F, 40).

A common thread for most participants was a desire for monitoring or check‐ins to provide accountability and encouragement for exercise engagement. For example, “I do think I need somebody to keep me going…if I know I've got to talk to somebody at the end of the fortnight or week to tell them what I've done that would motivate me to keep going…just a bit of a check in 'how are you going with the exercise” (P5, F, 65) and “I think if you've got somebody actually checking in on you then I think that's a good motivator” (P4, F. 42). Another participant, lacking in motivation, wished for a personal trainer in the ‘ideal world' and desired accountability: “Somewhere where I can't cheat…although I'm motivated now that I know I have to do something, I still need pushing, I think most people do. I don't mind being pushed and I like someone to monitor where I am” (P15, M, 61). The desire for accountability to review exercise progress was a common theme: “You need somebody even if it's just occasional to say how's it going, how's your progress because then you know you've got to progress because somebody's going to be asking you at some point” (P1, F, 69). Several participants also referred to the desire for goals that are challenging to foster motivation. For example, “It would need to be target‐driven and a challenge…something like the 6MWT if you've done 500 this week you get that to 600…to have weekly targets” (P17, M, 62) and “The one I like is the challenge and goals. I like the goals. I had a Fitbit once and I lost it but that kind of thing I would personally use without any intervention from anybody” (P11, F, 71) and “I would have to set myself a goal of doing a bit more each time and working up” (P9, F, 66). Further, participants that were more physically active tended to use exercise targets: “I have a Fitbit and I try and walk at least 10,000 steps a day, so that's my goal” (P2, F, 38) and using a Samsung watch “I use it for my heart rate…I try and keep it around 130” (P16, F, 40).

### 2.5 Self‐Presentation: Keeping up Appearances

This theme concerned self‐presentational concerns and exercise identity (i.e., not being the sporty type) and not wanting to appear acutely unwell or in poor physical condition. For example, the self‐consciousness associated with breathlessness hindered PA engagement: “My breathing is really bad so you feel like people are looking at you…it's the invisible illness side of PH really…you look fairly young, fairly okay. Why are you breathing like a 90‐year‐old…so that can be a bit of a barrier…maybe that's why I don't push myself as much as I should” (P8, F, 49). For another, fatigue caused some degree of anxiety as articulated in the first theme, however, the underpinning issue appeared to be about control over self‐presentation, and this is evident in a comparison of walking in an indoor pool versus walking outdoors:

When I'm walking outside and I get the tiredness I may have an element of panic thinking I'm going to have to sit on somebody's wall…but in the water it seems different…I don't get that panic feeling…I can just stop walking and lean on the side of the pool and nobody would think a thing about it…but if I was in the street walking I'd be reluctant to stop…I think It's the fact that if somebody looks at me being like that they'll see me as weak, old, decrepit and I don't want that.

(P11, F, 71)

Such self‐presentational concern related to exertion were not gender specific and echoed by men too. For example, “When you get breathless it's very hard to control that…other people around you don't understand it…why you're going so slowly or why you have to stop and that adds to the pressure…stress makes you more breathless” (P17, M, 62), and, “The most frustrating thing is people. When you can't walk because you're stalling for a breath…people will say ‘are you okay?’ I hate it ‘do you want a drink of water?’…No…maybe I should have a big sign on my head saying I've got PH‐ can't breathe properly. Leave me alone” (P12, M, 60). For others, self‐presentational concerns were related to the stigma of carrying portable oxygen to exercise: “I don't know whether to do it with or without oxygen. I don't like wearing oxygen…I don't like people looking at me with a big bag on my back…it's a bit of a stigma” (P15, M, 61) and “I have to have 15 h of oxygen a day…the other day we went for a walk…I didn't walk very far and I went back and sat in the car…I have an oxygen cylinder like a backpack and you just think I'm not going out with this. It puts me off…I wouldn't go and pick my grandchildren up with it” (P6, F, 70). Concerns related to self‐presentation appear to act as a barrier to exercise engagement for several participants with high self‐presentational concerns leading to either anxiety surrounding exercise engagement or avoidance of PA.

The salience of identity management was also apparent in the data with several participants that tended to be less physically active, identifying themselves as not the ‘sporty type'. For example, “I mean I've never been gym bunny” (P9, F, 66) and “I've never been over‐active like that” (P11, F, 71), and “I'm not a physically fit person I never have been” (P5, F, 65). Sometimes, participants were also derogatory of themselves in relation to PA: “I suppose if I'd been a keen sports person then I would be asking the right questions but because I haven't been, because I have been a bit of a slob when it comes to exercise that I'm probably not asking the right questions” (P1, F, 69) and “I've never been one for going to the gym or anything like that. I'm far too lazy to do things like that” (P15, M, 61). These quotes are primarily related to sport, fitness or the gym and indicate a narrow focus equating being physically active with participation in sports or attending a gym or leisure centre for structured exercise.

### 2.6 Little PA Advice: Patient‐Driven Communication

Most participants reported receiving very little in relation to PA advice or information. For example: “You go for the day, a CT scan, your bloods done and the walk test, then you meet the doctor who's treating you…there's no mention of you should walk everyday…no one has mentioned exercise” (P6, F, 70) and “There was quite a lot of information on certain things but nothing on exercise” (P17, M, 62); “They only talk about oh you've done well on your walking test and we'll see you in 3 months” (P12, M, 60).

Others reported receiving either inconsistent or unclear PA messaging: “I feel like the messaging on exercise has been quite inconsistent depending on different consultants…when I was first diagnosed (aged 13) it was don't do any sports, no exercise…Its always been quite wishy washy in terms of 'oh regular walking” (P2, F, 38) and “My doctor told my wife 'he needs to walk and get out and do things but in moderation' but there was no structured sheet or anything like that…it was just 'he needs to start moving, he can't just sit there” (P15, M, 61) and “It was exercise is good for you. Do it as much as you can until the point of symptom onset” (P21, M, 34).

This theme was underpinned by patient‐driven PA information in that PA information or advice was provided primarily when it was requested by the patient. For example, “It would be me bringing it up in consultant appointments…oh I do this, is that okay? It wouldn't be them saying 'what are you doing from a physical exercise standpoint? What are you doing, with what regularity, how strenuous…I wouldn't get those questions on my regular consultations” (P2, F, 38) and “When I was first diagnosed in 2013 I was taught by a physio some exercises for arms and legs…every day I used to do that…I had to request that because I was feeling so ill and weak…the point is it's never offered and you have to go out there and get it” (P4, F, 42). Others had sought out specialist exercise support for themselves: “Self‐referral. I heard through the sarcoidosis website that somebody else had been referred to this physio lung course so I made enquiries locally and got referred” (P10, F, 62) and “I actually made such an effort to contact people at the hospital in [……] to say I want to be on some sort of PH exercise and they said we don't have anything, it doesn't exist” (P7, F, 57).

Participants reported wanting to receive PA information and advice from their treating clinicians including content concerning the benefits of exercise and clear instruction on how to perform the behaviour. For example, “I think consultants need to encourage it…just say the benefits of it really…each time they go to an appointment, or have a leaflet to say 'look, this is what it can do” (P14, F, 33) and “I definitely think there should be information given on health and exercise. It should say 'this is what you need to do. If you do this, this is the benefit” (P17, M, 62). Time was recognised as a barrier to the provision of PA advice amongst clinicians but participants suggested a role for clear verbal encouragement of PA and signposting for further support and resources: “If the clinicians haven't got time then they could give you a leaflet or a website to get further information…they can say a one‐liner 'we recommend exercise, why don't you go here to look for it…I would go to the PHA website, they've got some great leaflets they send out. I don't think there's one on exercise” (P10, F, 62). Participants also desired to receive reassurance regarding the safety of exercise to avoid harm: “It's to know what's possible and what I can safely do and so on…you're left feeling that they don't know any more than you do and perhaps they don't” (P1, F, 69) and “What you need to hear is if you get breathless you can't make yourself worse and I think that's what I've been told. You've just got to push through as much as you can” (P17, M, 62). In addition to clarity on the benefits of exercise, clear guidance concerning the appropriate exercise intensity was also desired: “I have to have a reason for what I'm doing and is it going to be beneficial…how far can you push yourself? I mean I can get the figure for normal people not me” (P11, F, 71).

## 3 Discussion

To our knowledge, this is the first study in the UK to explore in depth, attitudes towards and the dimensions influencing PA engagement amongst those living with PH. The study found an interplay of dimensions that influenced PA engagement including fear and anxiety of breathlessness and overdoing it, lack of confidence, lack of motivation and self‐presentational concerns. The study also identified a lack of PA advice or information from treating clinicians and that where PA advice was provided, it tended to be patient driven.

A key novel contribution of the present study relates to self‐presentational concerns and exercise identity. Specifically, the visible symptoms of PH (e.g., embarrassment associated with breathlessness, physical condition, or oxygen requirements) hindered PA engagement because of the desire for a positive outward self‐presentation. Identity management also underpinned PA motivation with several participants identifying as ‘not the sporty type' or ‘gym bunny'. Such identity construction likely serves to protect self‐esteem by avoiding situations where one might appear less physically capable. This data also indicates a narrow conception of PA as denoting sports participation or attending a gym. Clearly more work is needed to convey the value of lifestyle‐based PA such was walking and to broaden patient conceptions about PA. The patients that do not identify as the ‘sporty' or ‘exercise' type are less likely to ask their treating clinician about PA and may not increase their PA if not encouraged to do so by their treating clinicians and hence the important role of PH clinicians in advocating PA.

Consistent with previous research [12, 14], the present study found that a lack of motivation (lack of self‐discipline) was the primary barrier to PA engagement in PH. Low levels of motivation underpinned the desire for PA monitoring or check‐ins to provide accountability and encouragement for exercise engagement. Similar findings concerning accountability and monitoring have been reported previously amongst those living with PH [16]. The present study also identified the value of targets or goals to mobilise PA efforts with some participants using smart wearables to keep to heart rate or step count targets. Interventions that target patient motivation and PA self‐regulation through goal setting, self‐monitoring and external review of behaviour would be valuable in this population, and preliminary evidence supports the utility of these techniques for increasing PA in PH [25]. Indeed, self‐monitoring and goal setting are evidence‐based behaviour change techniques to increase PA [26, 27].

The present study found that PA engagement was hindered by fear and anxiety of breathlessness, heart damage or overexertion and mirrors previous findings of those living with PH in Ireland [15]. Survey findings have also identified fatigue and dyspnoea to be common barriers to PA in PH [13]. However, the inductive approach of the present study extends previous findings in that fear and anxiety are intertwined with uncertainty regarding exercise ability and in some cases, participant inability to judge whether symptoms such as dyspnoea and fast heart rate are a reason to stop exercise (negative) or benign (positive‐ normal reaction to exercise and exertion) (i.e., uncertainty in interpreting heart rate/breathing rate as a normal response to exercise vs being a warning sign). Indeed, many participants struggled with anxiety and discussed how anxiety causes breathlessness and chest tightness leading to panic, with PA increasing anxiety due to the high interoceptive sensitivity of bodily sensations (heartbeat, breathing) on exertion, in addition to self‐presentational and motivational concerns highlighted previously.

A complex interplay of dimensions including anxiety, fear, uncertainty over capability, lack of enjoyment, and safety combine to hinder both motivation for and PA engagement in PH. These findings may help to illuminate the reasons underpinning a ‘lack of interest or will', a ‘lack of pleasure' and ‘lack of ability' towards PA in those living with PH found previously [12, 14]. The use of cognitive restructuring for fear of exertional dyspnoea and graded exposure to exercise are likely to be worthwhile techniques to alleviate anxiety and increase PA in those living with PH.

In contrast to previous findings [13] most participants in the present study did not recall receiving PA recommendations by their PH clinicians. Rather, PA advice or reassurance to exercise was provided primarily upon request (i.e., patient‐driven) and represents a new finding. Only one previous study has documented a lack of PA advice [16], and consistent with the present study, patient desire for exercise advice directly from their treating clinicians. Participants wanted treating clinicians to clarify the benefits of PA for PH in addition to providing reassurance concerning safety and guidance on appropriate exercise. Given the findings in relation to accountability and monitoring, clinicians could play a role in providing external accountability for patient PA efforts during outpatient appointments.

Several participants noted a lack of access to tailored exercise programmes such as pulmonary rehabilitation. Some had access to a physiotherapist, but provision appeared to be variable. Several participants had previously attended pulmonary rehabilitation but had transitioned to independent PA engagement following completion. Further work is needed to identify PA interventions that reduce the dominant exercise barriers and foster self‐regulation to support long‐term exercise adherence. Given the limited access to rehabilitation programmes, recent research has examined the effects of distance‐based PA interventions to improve health outcomes in PH. Meta‐analytic findings support the effectiveness of home‐based programmes compared to centre‐based programmes with comparable improvements in clinical outcomes [28]. There is promising research in this area promoting home‐based PA with remote support and/or wearable technology [25, 29, 30] but further research is needed to determine effectiveness in relation to sustained PA behaviour change and scalability.

## 4 Conclusions

To conclude, an interplay of dimensions influenced PA engagement including fear and anxiety of breathlessness and overexertion, lack of confidence, lack of motivation and self‐presentational concerns. The influence of self‐presentation and identity in exercise‐avoidance behaviours in PH is a novel finding. The study also found recollections of little PA advice from treating clinicians and a tendency for patient driven PA information. Future PA interventions that alleviate fear and anxiety through cognitive restructuring, provide reassurance on the safety and value of exercise, and include clear instruction on behaviour and graded exposure would be worthwhile to increase PA in PH. Interventions that broaden the conception of PA and promote lifestyle‐based PA alongside fostering patient motivation for PA through goal setting, self‐monitoring and review of behaviour are also likely to be valuable. Such interventions are likely to require the involvement of PH clinicians, particularly in the alleviation of fear and anxiety, provision of reassurance to exercise, and reaching those that do not have an exercise identity or history of PA engagement.

## 5 Study Limitations

Our study recruited participants in the UK; therefore, findings may not be generalisable. The potential for selection bias and recall bias are further limitations. Strengths of the study include a diverse, national sample, and capture of participants with varying health‐related quality of life scores with most (76%) having intermediate to high risk QoL scores.

## Author Contributions

Sarah J. Hardcastle conceived the original idea. Sarah J. Hardcastle, Ciara McCormack and Zachary Wells designed the protocol and collected the data. Zachary Wells transcribed some of the data. Sarah J. Hardcastle analysed the data and wrote the manuscript with support from all authors. All authors read and approved the final manuscript.

## Ethics Statement

This study followed the principles of the Declaration of Helsinki. Approval was granted by Sheffield Hallam University Research Ethics Committee (Reference ER50098142). Informed consent was obtained from all individual participants included in the study.

## Conflicts of Interest

The authors declare no conflicts of interest.

## Supporting information

Supporting File 1

Supporting File 2

## Data Availability

The data that support the findings of this study are available on request from the corresponding author. The data are not publicly available due to privacy or ethical restrictions.

## References

[1] M. Humbert , G. Kovacs , M. M. Hoeper , et al., “2022 ESC/ERS Guidelines for the Diagnosis and Treatment of Pulmonary Hypertension,” European Respiratory Journal 61 (2023): 2200879, 10.1183/13993003.00879-2022. 36028254

[2] C. Schmidt , M. Monteiro , I. Furtado , et al., “Physical Activity and Its Clinical Correlates in Chronic Thromboembolic Pulmonary Hypertension,” Pulmonary Circulation 12, no. 2 (2022): e12048, 10.1002/pul2.12048. 35514778 PMC9063965

[3] L. González‐Saiz , A. Santos‐Lozano , C. Fiuza‐Luces , et al., “Physical Activity Levels Are Low in Patients With Pulmonary Hypertension,” Annals of Translational Medicine 6, no. 11 (2018): 205, 10.21037/atm.2018.05.37. 30023368 PMC6035982

[4] Department of Health and S Care ., Physical activity guidelines: UK Chief Medical Officers' report (London: DHSC, 2019.

[5] F. C. Bull , S. S. Al‐Ansari , S. Biddle , et al., “World Health Organization 2020 Guidelines on Physical Activity and Sedentary Behaviour,” British Journal of Sports Medicine 54 (2020): 1451–1462. 33239350 10.1136/bjsports-2020-102955 PMC7719906

[6] L. A. Matura , H. Shou , J. S. Fritz , et al., “Physical Activity and Symptoms in Pulmonary Arterial Hypertension,” Chest 150 (2016): 46–56. 26892602 10.1016/j.chest.2016.02.633 PMC4980549

[7] S. Ulrich , M. Fischler , R. Speich , and K. E. Bloch , “Wrist Actigraphy Predicts Outcome in Patients With Pulmonary Hypertension,” Respiration 86 (2013): 45–51. 23234873 10.1159/000342351

[8] J. Minhas , H. Shou , S. Hershman , et al., “Physical Activity and Its Association With Traditional Outcome Measures in Pulmonary Arterial Hypertension,” Annals of the American Thoracic Society 19, no. 4 (2022): 572–582, 10.1513/AnnalsATS.202105-560OC. 34473938 PMC8996274

[9] A. L. Albanaqi , G. R. M. Rahimi , and N. A. Smart , “Exercise Training for Pulmonary Hypertension: A Systematic Review and Meta‐Analysis of Randomized Controlled Trials,” Biological Research for Nursing 23, no. 3 (2021): 442–454. 33371736 10.1177/1099800420982376

[10] X. Zeng , H. Chen , H. Ruan , X. Ye , J. Li , and C. Hong , “Effectiveness and Safety of Exercise Training and Rehabilitation in Pulmonary Hypertension: A Systematic Review and Meta‐Analysis,” Journal of Thoracic Disease 12, no. 5 (2020): 2691–2705. 32642177 10.21037/jtd.2020.03.69 PMC7330286

[11] R. Glockl , T. Schneeberger , T. Boeselt , et al., “Exercise Training in Patients With Pulmonary Hypertension: A Systematic Review and Meta‐Analysis,” Pneumologic 73, no. 11 (2019): 677–685. 10.1055/a-1005-8678 31715636

[12] T. M. Cascino , V. V. McLaughlin , C. R. Richardson , et al., “Barriers to Physical Activity in Patients With Pulmonary Hypertension,” Pulmonary Circulation 9, no. 2 (2019): 1–8, 10.1177/2045894019847895. PMC6540505 30983524

[13] K. S. W. Chia , K. Brown , E. Kotlyar , et al., “Tired, Afraid, Breathles.' An International Survey of the Exercise Experience for People Living With Pulmonary Hypertension,” Pulmonary Circulation 10, no. 4 (2020): 2045894020968023, 10.1177/2045894020968023. 33240490 PMC7675876

[14] L. N. G. Lima , V. Veronez , P. R. A. Mendes , T. A. Kiyota , M. M. Moreira , and M. C. Pereira , “Relationships That Perceived Barriers to Physical Activity Have With Functional Capacity and Quality of Life in Patients with Pulmonary Hypertension,” Jornal Brasileiro de Pneumologia: Publicacao Oficial da Sociedade Brasileira de Pneumologia e Tisilogia 51, no. 1 (2025): 20240195, 10.36416/1806-3756/e20240195. PMC12097729 40172409

[15] C. McCormack , S. Cullivan , B. Kehoe , et al., “It Is the Fear of Exercise That Stops Me”—Attitudes and Dimensions Influencing Physical Activity in Pulmonary Hypertension Patients,” Pulmonary Circulation 11, no. 4 (2021): 1–9, 10.1177/20458940211056509. PMC8573491 34777786

[16] C. McCormack , B. Kehoe , S. Cullivan , et al., “Exploration of Physical Activity Knowledge, Preferences and Support Needs Among Pulmonary Hypertension Patients,” PLoS One 18, no. 1 (2023): e0277696, 10.1371/journal.pone.0277696. 36652433 PMC9847985

[17] O. Sangha , G. Stucki , M. H. Liang , A. H. Fossel , and J. N. Katz , “The Self‐Administered Comorbidity Questionnaire: A New Method to Assess Comorbidity for Clinical and Health Services Research,” Arthritis Care & Research 49 (2003): 156–163. 12687505 10.1002/art.10993

[18] J. Yorke , P. Corris , S. Gaine , et al., “EmPHasis‐10: Development of a Health‐Related Quality of Life Measure in Pulmonary Hypertension,” European Respiratory Journal 43 (2013): 1106–1113. 24232702 10.1183/09031936.00127113 PMC3971119

[19] B. C. O'Brien , I. B. Harris , T. J. Beckman , D. A. Reed , and D. A. Cook , “Standards for Reporting Qualitative Research: A Synthesis of Recommendations,” Academic Medicine 89, no. 9 (2014): 1245–1251, 10.1097/ACM.0000000000000388. 24979285

[20] V. Braun and V. Clarke , “Reflecting on Reflexive Thematic Analysis,” Qualitative Research in Sport, Exercise and Health 11, no. 4 (2019): 589–597, 10.1080/2159676X.2019.1628806.

[21] V. Braun and V. Clarke , Successful Qualitative Research: A Practical Guide for Beginners (Thousand Oaks: Sage Publications, 2013).

[22] V. Braun and V. Clarke , “Using Thematic Analysis in Psychology,” Qualitative Research in Psychology 3, no. 2 (2006): 77–101, 10.1191/1478088706qp063oa.

[23] S. J. Hardcastle and M. Caraher , “The Role of Foodbanks in the Context of Food Insecurity: Experiences and Eating Behaviours Amongst Users,” Appetite 105208 163 (2021): 105208, 10.1016/j.appet.2021.105208. 33774137

[24] S. Hardcastle and M. S. Hagger , “You Can't Do It on Your Own: Experiences of a Motivational Interviewing Intervention on Physical Activity and Dietary Behaviour,” Psychology of Sport and Exercise 12, no. 3 (2011): 314–323, 10.1016/j.psychsport.2011.01.001.

[25] C. McCormack , B. Kehoe , S. Cullivan , et al., “Safety, Feasibility and Effectiveness of the Remotely Delivered Pulmonary Hypertension and Home‐Based (PHAHB) Physical Activity Intervention,” ERJ Open Research 10, no. 1 (2024): 00608‐2023, 10.1183/23120541.00608-2023. 38264149 PMC10805266

[26] G. B. Samdal , G. E. Eide , T. Barth , G. Williams , and E. Meland , “Effective Behaviour Change Techniques for Physical Activity and Healthy Eating in Overweight and Obese Adults; Systematic Review and Meta‐Regression Analyses,” International Journal of Behavioral Nutrition and Physical Activity 14, no. 1 (2017): 42, 10.1186/s12966-017-0494-y. 28351367 PMC5370453

[27] S. Michie , C. Abraham , C. Whittington , J. McAteer , and S. Gupta , “Effective Techniques in Healthy Eating and Physical Activity Interventions: A Meta‐Regression,” Health Psychology 28, no. 6 (2009): 690–701, 10.1037/a0016136. 19916637

[28] I. C. Ribeiro , S. M. Sieczkowska , R. Jashchenko , et al., “Effectiveness and Safety of Home‐Based Versus Centre‐Based Exercise Programmes for Pulmonary Hypertension: A Systematic Review With Meta‐Analysis,” European Respiratory Review 34, no. 177 (2025): 250102, 10.1183/16000617.0102-2025. 40903048 PMC12406019

[29] A. S. Babu , R. Padmakumar , K. Nayak , R. Shetty , A. K. Mohapatra , and A. G. Maiya , “Effects of Home‐Based Exercise Training on Functional Outcomes and Quality of Life in Patients With Pulmonary Hypertension: A Randomized Clinical Trial,” Indian Heart Journal 71, no. 2 (2019): 161–165, 10.1016/j.ihj.2019.03.002. 31280830 PMC6620412

[30] L. Butāne , L. Spilva‐Ekerte , A. Skride , and D. Šmite , “Individually Tailored Remote Physiotherapy Program Improves Participation and Autonomy in Activities of Everyday Life Along with Exercise Capacity, Self‐Efficacy, and Low‐Moderate Physical Activity in Patients With Pulmonary Arterial Hypertension: A Randomized Controlled Study,” Medicina (Kaunas, Lithuania) 58, no. 5 (2022): 662, 10.3390/medicina58050662. 35630079 PMC9147937

