# Supplementary material for: Factors Influencing Physical Activity Engagement Amongst Those Living With Pulmonary Hypertension in the UK
Source: Pulm Circ. 2026 Jun 11;16(2):e70332. doi: 10.1002/pul2.70332 (PMC13254823; doi:10.1002/pul2.70332)
Supplement: Supplementary file 2 — Supporting File 2 [file PUL2-16-e70332-s001.docx]

**Supplemental File 2: Interview Guide**

| Introduction | |
| --- | --- |
| Thank you very much for agreeing to participate in this interview.  Today, we are going to talk about your experience of living with Pulmonary Hypertension and your physical activity behaviour.  We would like you to be as honest as possible during this interview, there is absolutely no right or wrong answers!  Also, if it is ok with you, we will audio-tape our discussion so that we can go back and listen to it at a later date for analysis. No one else will hear this tape and we will keep your personal information absolutely confidential and anonymous.  You may end the interview at any point, should you not want to continue.  Do you have any questions before I start the audio-recording? | |
| Questions | Prompts |
| 1. Could you say a few words about how important or not physical activity is to you? | **Prompt in what ways (physical health, mental health etc)** |
| 1. Could you describe your physical activity engagement prior to been diagnosed with PH (or in the past/your exercise history) | **How much, how often, how hard, what types** |
| 1. And what about your current physical activity participation? What does a typical week look like for you? | **How much, how often, how hard, what types/ask if they know what the physical activity guidelines are?**  **Aerobic, Strength/Resistance** |
| 1. What are the main influences on your physical activity? | **Facilitating factors.** |
| 1. What are the main barriers to physical activity/increased exercise participation? | **What prevents you from been physical active?**  **What else?** |
| 1. What would motivate you to increase your level of physical activity? | **What would help you to engage more or become more active** |
| 1. Have you received any advice or support from your PH team regarding physical activity and staying healthy? | **Either from nurses, PH clinicians**  **If yes, what was the specific advice? Has rehabilitation been offered?** |
| 1. What would be the most effective way of helping you to be more active, what would work for you? | **Exercise programs – Type, mode, support**  **Try gain understanding of the components they would like** |
| 1. In an ideal world, what would the perfect exercise program look like for you? | **What would be the important features? Level of contact/mode of delivery.** |
